# Longitudinal Trajectories of Cognitive Function Among Chinese Middle-Aged and Older Adults: The Role of Sarcopenia and Depressive Symptoms

**DOI:** 10.3390/brainsci15040408

**Published:** 2025-04-17

**Authors:** Shengxuan Jin, Jianqian Chao, Qian Jin, Beibei Yang, Gangrui Tan, Leixia Wang, Yanqian Wu

**Affiliations:** 1 Health Management Research Center, School of Public Health, Southeast University, Nanjing 210009, China; 2 School of Education Science, Qingdao University, Qingdao 266071, China

**Keywords:** cognitive trajectory, sarcopenia, depressive symptoms, longitudinal study, mediation analysis

## Abstract

**Objectives:** The longitudinal relationship between sarcopenia, depression, and cognitive impairment has been insufficiently studied in China. This study aimed to characterize the association between sarcopenia and cognitive impairment and the mediating role of depression using nationally representative data. **Methods:** 7091 middle-aged and older adults were analyzed from the China Health and Retirement Longitudinal Study (CHARLS) across three waves (2011, 2013, and 2015). Cognitive trajectories were modeled using a group-based trajectory model (GBTM), while multivariable ordinal logistic regression was employed to evaluate the associations with cognitive trajectories. The mediating role of depressive symptoms was assessed through bootstrap mediation analysis and cross-lagged panel modeling (CLPM). **Results:** Trajectory analysis identified four distinct cognitive function patterns: “High and Stable” trajectory (*n* = 2563, 36.73%), “Middle and Stable” group (*n* = 2860, 38.76%), “Middle and Decline” group (*n* = 1280, 18.62%), and “Low and Decline” group (*n* = 388, 5.90%). Sarcopenia and depressive symptoms were associated with the “Low and Decline” trajectory of cognitive function [Overall: OR (95%CI) of 0.315 (0.259, 0.382) and 0.417 (0.380, 0.459)]. Mediation analysis indicated that depressive symptoms accounted for 11.78% of the relationship between sarcopenia and cognitive trajectories. The cross-lagged panel modeling demonstrated a significant mediation pathway of “T1 cognitive function → T2 depression → T3 sarcopenia”, with T2 depression mediating 5.31% of the total effect. **Conclusions:** Our study identified four distinct cognitive trajectories, with sarcopenia and depressive symptoms significantly associated with worse cognitive trajectories over time. Depressive symptoms mediated the relationship between sarcopenia and cognitive function. This highlights the importance of integrating mental health and physical health interventions to address the interconnected risks associated with aging.

## 1. Introduction

Cognitive impairment represents a significant public health challenge worldwide, characterized by a range of cognitive function symptoms, including memory difficulties, executive dysfunction, language or visuospatial challenges, and changes in behavior [1]. Many seniors face the risk of spending their “golden years” struggling with cognitive impairment, which can range from mild cognitive impairment (MCI) to more severe forms of dementia, such as Alzheimer’s disease (AD), vascular dementia, and Lewy body dementia. A recent meta-analysis indicated that the global pooled prevalence of mild cognitive impairment (MCI) is approximately 19.7% (95% Confidence Interval: 18.3–21.1%), with time trends showing marked increases post-2019 (32.1%) relative to 19.8% between 2010 and 2018 and 14.5% before 2009 [2]. Furthermore, another systematic review of community-dwelling older adults found that overall CI prevalence ranged from 5.1% to 41% (median of 19.0%), with the median age-stratified prevalence rates of 12% for ages 50–59, 20.1% for ages 60–69, and 19% for those aged 70 and above [3]. In Asia, prevalence estimates among older adults range from 10.7% to 36% [4]. Cognitive function generally declines in older age, influenced by age, gender, lifestyle, and comorbid conditions [5]. As global populations age, the burden of cognitive impairment is expected to increase, with profound implications for families, healthcare systems, and social services. These epidemiological data underscore the urgent need for the early identification and management of modifiable risk factors for cognitive impairment [6].

Three interrelated conditions have emerged as critical factors in the cognitive health of aging populations: sarcopenia, depressive symptoms, and cognitive impairment. Sarcopenia—a geriatric syndrome characterized by an age-related loss of skeletal muscle mass, strength, and function—has been associated not only with physical disability but also with declines in cognitive function [7,8]. For instance, biological studies and epidemiological data suggest that reduced muscle function is associated with later-life cognitive impairment [9]. Hu et al. (2022) reported that elderly individuals with possible sarcopenia or sarcopenia had a higher risk of developing new-onset MCI [10], a finding that is echoed by international data [11]. Although systematic reviews have consistently shown associations between sarcopenia and cognitive impairment such as AD dementia, mild cognitive impairment, and rates of cognitive decline [12,13,14,15,16], the predominance of cross-sectional studies limits our ability to infer causality and fully understand the underlying mechanisms.

In parallel, depression, a common mental health disorder among older adults characterized by a prolonged depressed mood or loss of interest in activities [17], has been linked to both sarcopenia and cognitive impairment. Several studies, particularly from Asian populations, have demonstrated that higher depressive symptom scores are associated with an increased risk of sarcopenia [18,19,20,21], as supported by meta-analyses of cross-sectional data [22]. However, the causal direction remains controversial, as it is uncertain whether depressive symptoms contribute to the development of sarcopenia or if they emerge as complications arising from declining muscle and physical condition [23]. Moreover, depression is well recognized as both a risk factor and an early marker of cognitive decline [24]. Symptoms of depression are associated with impairments in neurocognitive functions, particularly those regulated by frontosubcortical networks, encompassing processing speed, attention, and executive functions [25].

Given the well-documented relationships, it is plausible that depression mediates the influence of sarcopenia on cognitive impairment [26]. Preliminary studies have supported the mediating role of depressive symptoms [27,28]. However, most have employed retrospective or cross-sectional designs, which limit causal inference. Moreover, the possibility of a bidirectional mediating mechanism involving depressive symptoms remains insufficiently explored in the current literature. To address these gaps, our study focuses on three primary objectives: First, we aimed to identify distinct trajectories of cognitive function in Chinese middle-aged and older adults based on data from the China Health and Retirement Longitudinal Study (CHARLS) and evaluate the impact of sarcopenia and depression symptoms on these cognitive trajectories. Second, we investigated the bidirectional relationship between sarcopenia and cognitive function using cross-lagged modeling. Third, we specifically examined the mediating relationship of depressive symptoms between sarcopenia and cognitive function, revealing the dynamic interactions among these three factors. This study seeks to clarify these complex associations and inform early intervention strategies and public health policies designed to mitigate the growing burden of cognitive impairment.

## 2. Method

### 2.1. Participants and Procedure

This study draws on data from the China Health and Retirement Longitudinal Survey (CHARLS), a nationally representative longitudinal study targeting adults aged 45 and older in mainland China. Designed to track the dynamics of aging, health, retirement, and economic well-being, CHARLS initially surveyed 17,708 participants from 10,257 households across 150 counties in 28 provinces, employing a four-stage, stratified cluster sampling method to achieve a representative sample. Baseline data collection occurred in 2011–2012, with follow-up waves in 2013, 2015, 2018, and 2020. Comprehensive information on socioeconomic status, lifestyle behaviors, health conditions, and functional status was obtained in each wave [29]. Prior to their participation in the study, all participants provided informed consent. The data collection for CHARLS was granted ethical approval by the Biomedical Ethics Review Committee of Peking University (reference number IRB00001052-11015). Only those who had given written informed consent were eligible for inclusion in the study.

**Figure 1:**
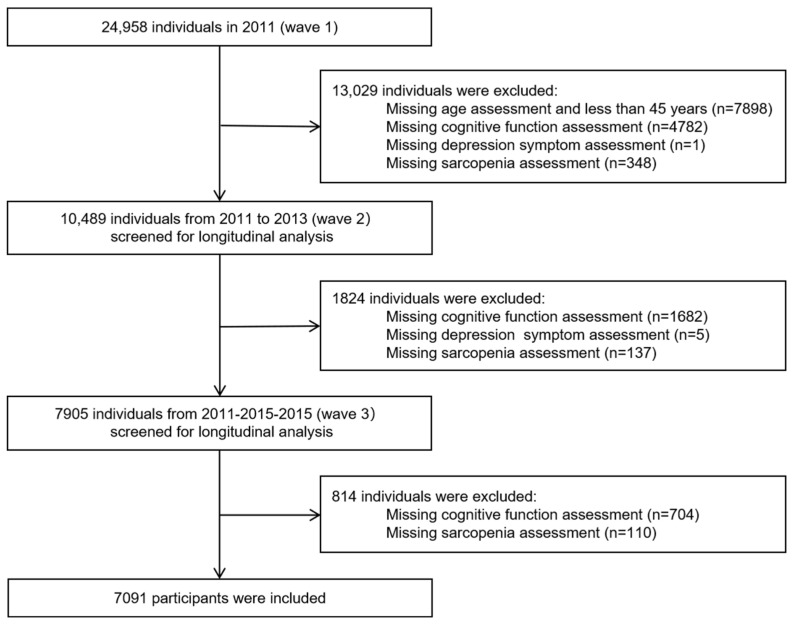
Flowchart of participant selection from the China Health and Retirement Longitudinal Study (CHARLS).

As the standardized measurements of sarcopenia in CHARLS were only available from 2011 (wave 1), 2013 (wave 2), and 2015 (wave 3), these three waves were chosen for this study. The included criteria were (1) age ≥45 years at baseline, (2) no information on cognitive impairment assessment, (3) no information on sarcopenia assessment, and (4) no information on depression symptoms assessment. The detailed flowchart of the sample selection process is shown in Figure 1.

The sample size of cross-sectional study participants calculated from Kendall’s sample estimation should be 5–10 times that of the independent variable [30]. The sample size of this study meets that requirement.

### 2.2. Measurements

**Sarcopenia** was assessed in accordance with the criteria proposed by the Asian Working Group for Sarcopenia (AWGS) 2019, which include muscle strength, appendicular skeletal muscle mass (ASM), and physical performance [31]. Measurements of handgrip strength (in kilograms) were obtained for both the dominant and non-dominant hand, with participants instructed to apply maximum force. For each participant, duplicate tests were performed on both hands, with the dynamometer held at a vertical angle (90°). The mean of the recorded peak strength values was used. In cases where measurement of one hand was not feasible, the highest value from the other hand was assessed. A low grip strength threshold of less than 28 kg for men and less than 18 kg for women has been established according to the AWGS 2019 guidelines.

The ASM for the Chinese cohort was approximated using a previously reported anthropometric formula [32]:

ASM=0.193×weight+0.107×height−4.157×gender−0.037×age−2.631

Height, weight, and age were recorded in centimeters, kilograms, and years, respectively. Gender was coded as 1 for males and 2 for females.

The threshold for low muscle mass was based on the lowest 20th percentile of height-adjusted muscle mass (ASM/height^2^) within the study sample, specific to each sex, which was <4.89 kg/m^2^ for women and <6.79 kg/m^2^ for men [33]. Sarcopenia is defined as low muscle mass coupled with low muscle strength or low physical performance.

**Cognitive function** was assessed in two distinct domains: episodic memory and mental intactness [34]. Episodic memory is susceptible to early cognitive decline and is considered a hallmark of conditions such as mild cognitive impairment and dementia. At the same time, mental intactness captures a range of cognitive processes (e.g., numerical ability, time orientation, and visuospatial skills) that are essential for daily functioning and reflect broader cognitive integrity. The assessment of episodic memory entails the presentation of a list of ten Chinese nouns to the participants, who are then required to recall them in any sequence (immediate recall) and subsequently after a four-minute interval (delayed recall). The episodic memory score is derived from the mean of the immediate and delayed recall scores, with a potential range of 0 to 10. Mental intactness was assessed using three tests: numerical ability, time orientation, and figure drawing. The numerical ability test consisted of serial subtraction of 7 from 100, repeated five times. The time orientation assessment required participants to provide the current date (month, day, year), the day of the week, and the season. For the figure drawing task, participants were asked to replicate an image depicting two overlapping pentagons. A score of one point was awarded for each correct answer or successfully reproduced image, with a maximum score of 11 for mental intactness. Total cognitive scores are calculated as the sum of episodic memory and mental intactness scores and range from 0 to 21, with higher scores indicating better cognitive function [35].

**Depressive symptoms** The Center for Epidemiological Studies Depression Scale (CES-D-10) was used to measure depressive symptoms in the CHARLS questionnaire. This scale aims to quantify the severity and frequency of self-reported depressive symptoms in the community, distinct from clinical depression diagnosis [36]. Among older adults participating in the CHARLS study, it exhibited strong validity and reliability [37]. The respondents were requested to evaluate the frequency of occurrence of the ten depressive symptoms listed in the 10-item CES-D over the preceding week. Scoring for each item was conducted in accordance with a four-point Likert scale, ranging from 0 (indicating “rarely” or “never”) to 3 (“most” or “all of the time”), resulting in a potential cumulative score between 0 and 30. Prior to data analysis, two positively worded items underwent reverse coding. The elevated scores signify a greater severity of depressive symptoms. A cutoff score ≥10 was used to identify the respondents who had significant depressive symptoms [38,39].

### 2.3. Potential Covariates

Previous studies have identified the correlations between age, gender, education, lifestyle, BMI, functional abilities, and cognitive scores [6,23,40]. Consequently, the present study incorporated the following sociodemographic and health-related characteristics included in the analyses: age, gender, residence, educational level, marital status, health insurance, sleep, smoke, drink, body mass index (BMI), number of chronic diseases, Activities of Daily Living (ADL), Instrumental Activities of Daily Living (IADL), pain, and self-reported health (SRH). Participants were stratified by age into three groups: 45–59, 60–74, and 75 years or older. Gender was classified as male or female. Residence was divided into rural and urban settings. Educational attainment was categorized into three levels: primary school or below, junior high school, and senior high school or above. Marital status was classified as either married or other. Health insurance coverage, as well as smoking and drinking status, were each recorded as either “yes” or “no”. Sleep was reported as the mean with its standard deviation (SD). BMI was classified into four categories: normal, underweight, overweight, and obese, as calculated by weight (kg)/(height (m) × height (m)). Chronic disease counts were grouped as none, one, or two or more. Functional abilities were further assessed using ADL and IADL by the Katz ADL scale and the Lawton IADL scale. Questionnaires were answered in four categories: (1) No, I don’t have any difficulty; (2) I have difficulty but can still do it; (3) Yes, I have difficulty and need help; and (4) I cannot do it. In this study, participants selecting either of the latter two options for any item were classified as ADL- or IADL-dependent. Pain was assessed based on the question: “On what part of your body do you feel pain? Please list all parts of body you are currently feeling pain”. Respondents were categorized as ‘no’ if they reported no pain in any part of the body and ‘yes’ if pain was present. SRH was assessed on a five-level scale: very good, good, normal, poor, and very poor.

### 2.4. Statistical Analyses

Categorical variables are expressed as *n* (%) and continuous variables as mean ± standard deviation (SD). The Pearson chi-squared and *t*-test/ANOVA were used to compare the characteristics of the different trajectory groups at baseline.

Group-based trajectory modeling (GBTM) was employed to explore the heterogeneity of cognitive function trajectories on the longitudinal data. GBTM, as a potential class growth model, identifies distinct trajectory groups and estimates the shape of their cognitive function trajectories over time [41]. The model selection was an iterative testing process to determine the optimal number of trajectory groups. Criteria for model adequacy included statistical measures (the *p*-values of model parameters and the confidence intervals of trajectory estimates), visual inspection of predicted trajectories, Bayesian Information Criterion (a smaller BIC indicating a better model fit), and the average posterior probability (AvePP; above 0.7 indicated optimal fit) [42]. Multivariable ordinal logistic regression models were employed to examine the associations of sarcopenia and depressive symptoms with cognitive trajectories. Odds ratios (ORs) and 95% confidence intervals (CIs) were calculated to quantify the impact of sarcopenia and depression on cognitive function trajectories. Additionally, subgroup analyses were conducted by age and gender to explore potential variations in these associations across demographic groups, ensuring the robustness and relevance of findings within distinct population strata.

To investigate the mediation effects of depressive symptoms on the relationship between sarcopenia and cognitive trajectories, a mediation analysis was conducted using a bootstrap approach in R, facilitating the robust estimation of mediation paths. A non-parametric bootstrap procedure with 5000 resamples was utilized. Following the mediation analysis, cross-lagged panel modeling (CLPM) was employed to examine the bidirectional relationships between sarcopenia and cognitive function across three time points, providing insights into the temporal dynamics between these variables. Model estimation was performed using maximum likelihood with robust standard errors (MLR). Model fit was assessed through established criteria: a root mean square error of approximation (RMSEA) and standardized root mean square residual (SRMR) of <0.08, and a comparative fit index (CFI) and Tucker–Lewis index (TLI) ≥ 0.90, indicating an acceptable model fit [43].

Statistical analyses were performed using R 4.4.0 (R core team, Wien, Austria), Stata version 18.0 (StataCorp, College Station, TX, USA), and Mplus 8.0. A two-sided *p*-value < 0.05 was used to determine statistical significance.

## 3. Results

### 3.1. Cognitive Trajectory Modeling

**Figure 2:**
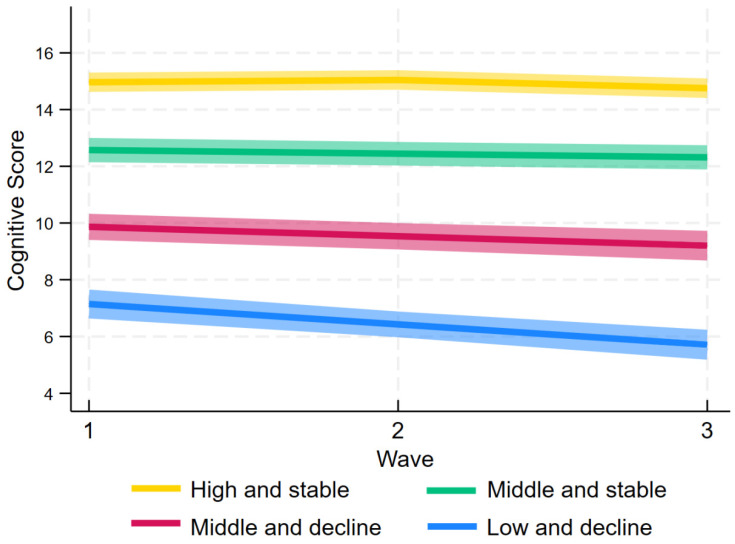
Trajectories of cognitive function scores by GBTM.

The fitted parameters for cognitive impairment trajectory groups among study participants by GBTM are presented in Table 1. The analysis indicates that under the criterion of AvePP higher than 0.70, the model with four distinct trajectory groups provides the best fit, with the smallest BIC and AIC results of −51,161.54 and −51,116.91. The AvePP values for groups 1–4 were 0.85, 0.79, 0.76, and 0.85, respectively. These groups, shown in Figure 2, include “High and Stable” trajectory (36.73%), “Middle and Stable” group (38.76%), “Middle and Decline” group (18.62%), and “Low and Decline” group (5.90%). Univariate analyses of trajectories with baseline characteristics and symptoms, as shown in Table 1 and Table 2, identified significant differences in baseline variables, symptom prevalence, and scores across trajectories, with the exception of health insurance status (*p* < 0.05).

### 3.2. Descriptive Statistics

The baseline characteristics of the overall sample (*n* = 7091) in Table 2 reveal a predominantly middle-aged population, with 58.6% aged 45–59 and 38.3% aged 60–74. Males constituted 54.0% of the sample, and the majority (75.5%) resided in rural areas. Educational attainment was generally low, with 58.3% having a primary school education or less, and 91.2% of participants were married. Medical insurance coverage was extensive (94.8%), while 43.3% reported smoking and 37.2% drinking. Regarding BMI, 50.9% were classified as normal weight, with 32.4% overweight. Health assessments indicated that 39.0% had two or more chronic diseases, and self-rated health was reported as good or very good by 25.6% of the participants, with 19.6% reporting poor health. Functional limitations were present in 12.8% for ADL and 14.7% for IADL, while 30.2% reported pain.

Table 3 summarizes sarcopenia, depressive symptoms, and cognitive function scores (MMSE) across different waves. The prevalence of sarcopenia in the study population showed a slight increase, from 5.3% in 2011 to 6.5% in 2015. Mean depressive symptom scores remained relatively stable across waves, with scores of 7.60 ± 5.98 in 2011, 7.31 ± 5.46 in 2013, and 7.44 ± 6.08 in 2015. The proportion of participants reporting depressive symptoms was consistent over time, with 31.6% in 2011, 28.0% in 2013, and 30.4% in 2015. Mean cognitive scores, measured by MMSE, demonstrated a slight decline from 12.62 ± 3.10 in 2011 to 12.23 ± 3.28 in 2015, reflecting a gradual reduction in cognitive function over this period. The results were statistically significant between waves (*p* < 0.05).

### 3.3. Association Between Sarcopenia and Depressive Symptoms with Cognitive Trajectories

**Figure 3:**
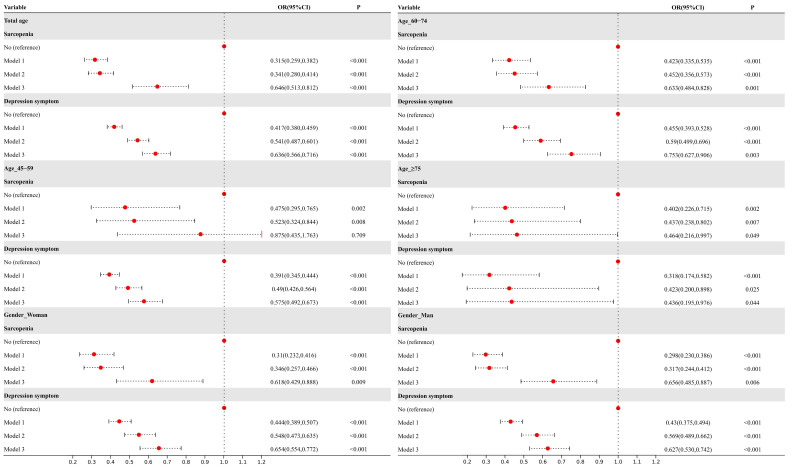
Association between sarcopenia and depression symptoms with trajectories of cognitive scores. Note: Model 1 includes sarcopenia and depression symptom variable. Model 2 adjusted for health-related variables (number of chronic diseases, ADL, IADL, pain, and SRH). Model 3 further adjusted for age, gender, residence, educational level, marital status, health insurance, sleep, smoke, drink, and BMI.

Figure 3 shows the results of multivariable ordinal logistic regression analyses of the relationship between sarcopenia, depression symptoms, and cognitive trajectories. The total population and stratified subgroups (by gender and age) were conducted. Three regression models were analyzed: Model 1 included only sarcopenia and depressive symptoms, Model 2 additionally included health-related variables, and Model 3 further controlled for demographic characteristics. Only the odds ratios (ORs) for sarcopenia and depressive symptoms are presented in the figure.

In the overall population, older adults with sarcopenia and depressive symptoms were more likely to belong to the “Low and Decline” cognitive trajectory group [Model 1: OR (95%CI) of 0.315 (0.259, 0.382) and 0.417 (0.380, 0.459), respectively]. Upon incremental adjustment for health and demographic variables, the ORs increased.

Subgroup analyses by age and gender revealed patterns consistent with the overall population. The propensity for sarcopenia and depression was higher in the “Low and Decline” group for those aged ≥75 [Model 1: OR (95%CI) of 0.402 (0.226, 0.715) and 0.318 (0.174, 0.582), respectively], followed by those aged 60–74. For gender, men had a higher risk value [Model 1: OR (95%CI) of 0.298 (0.230, 0.386) and 0.430 (0.375, 0.494), respectively], although the risk difference between males and females was minimal.

### 3.4. Mediation Effect of Depression Symptoms Between Sarcopenia and Cognitive Trajectories

The baseline mediation analysis (Supplementary Table S1) shows that the indirect effect of sarcopenia on cognitive trajectories via depressive symptoms was significant (*β*= −0.0739; 95% CI: −0.0961, −0.0500). The total effect of sarcopenia on cognitive trajectories is −0.6273 (95% CI: −0.7220, −0.5300), indicating a significant mediation effect of depressive symptoms that accounts for 11.78% of the total effect.

**Figure 4:**
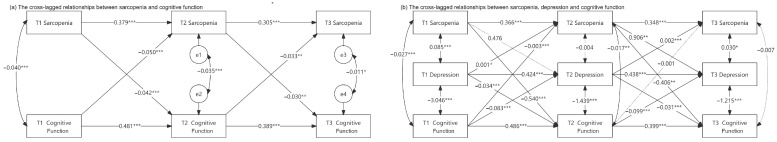
The longitudinal mediating effect of depressive symptoms on the relationship between sarcopenia and cognitive function by CLPM. Note: The insignificant paths are indicated by dotted lines. * indicates *p* value < 0.05; ** indicates *p* value < 0.01; *** indicates *p* value < 0.001.

To further explore these relationships, the CLPM analysis was conducted. Significant correlations (*p* < 0.05) are observed among sarcopenia, depression scores, and cognitive function scores (Supplementary Table S2). Figure 4 illustrates the cross-lagged relationships between sarcopenia and cognitive function (a) and the mediating role of depression (b), both adjusted for covariates, including gender, age, and educational level. The results indicate a bidirectional, lagged negative effect between sarcopenia and cognitive function after covariate adjustment. Specifically, sarcopenia at T1 significantly predicts cognitive function at T2 (*β* = −0.042, *p* < 0.05), and sarcopenia at T2 predicts cognitive decline at T3 (*β* = −0.030, *p* < 0.05). Conversely, cognitive impairment at T1 predicts increased sarcopenia risk at T2 (*β* = −0.050, *p* < 0.05), and cognitive function at T2 predicts sarcopenia at T3 (*β* = −0.033, *p* < 0.05). The unadjusted model is shown in Supplementary Figure S1, and model fit indices are reported in Supplementary Table S3.

One significant mediation pathway is observed in “T1 cognitive function → T2 depression → T3 sarcopenia” that cognitive impairment at T1 significantly predicts increased depressive symptoms at T2 (*β* = −0.083, *p* < 0.05). Depressive symptoms at T2 predict greater sarcopenia at T3 (*β* = 0.002, *p* < 0.05). The indirect effect is statistically significant, accounting for 5.31% of the total effect (95% CI: −0.0056 to −0.0014), as shown in Supplementary Table S1.

### 3.5. Sensitivity Analyses

Given the stability of the findings, sensitivity analyses were performed. First, trajectory analyses were performed on episodic memory and mental intactness scores to validate the number of trajectories. The results showed the best fit for all four groups. The fitting procedure and graphs are in Supplementary Table S4 and Figure S2. Second, a repeated-measures mixed model was used to test the associations of sarcopenia and depression as predictors (separately), with total cognitive score as the outcome. The results revealed sarcopenia (*β* = −0.018, *z* = −0.10, *p* = 0.922) and sarcopenia × time interaction (*β* = −0.537, *z* = −6.95, *p* = < 0.001) and depression (*β* = −0.740, z = −8.05, *p* < 0.001) and Depression × Time interaction (*β* = −0.212, *z* = −6.37, *p* = < 0.001). The results of the effects of including covariates were consistent.

## 4. Discussion

This study examined 7091 middle-aged and older adults across three waves to investigate the relationship between baseline sarcopenia, depression, and cognitive trajectories, as well as the mediating role of depressive symptoms. The results identified four cognitive trajectories in this population, and we demonstrated that sarcopenia and depression symptoms were associated with worse cognitive performance trajectories over time. A bidirectional relationship between sarcopenia and cognitive function was also revealed through cross-lagged analyses, suggesting that these two symptoms may interact with each other over time. Mediation analyses using bootstrapping indicated that depressive symptoms mediated the effect of sarcopenia on cognitive trajectories. Additionally, CLPM analysis demonstrated a pathway whereby T1 cognitive function led to T2 depression, which, in turn, contributed to T3 sarcopenia. Our findings highlight the importance of incorporating cognitive function, physical performance, and mood assessments in routine clinical evaluations for middle-aged and older adults. Early identification of sarcopenia and depressive symptoms may provide a critical opportunity for interventions to prevent or slow cognitive decline.

Four cognitive trajectories were identified in our study, and there were relatively minimal differences in the slopes between the four trajectory groups. The present trajectories are consistent with those identified by Xu et al. and Gardeniers et al. [44,45], aligning with prior research that reported trajectories ranging from 2 to 5. The disparity in the cognitive trajectories can be attributed to the use of distinct populations and trajectory models across different research studies [46]. The “High and Stable” trajectory, observed in 36.1% of Chinese older adults, represents an ideal outcome, reflecting consistently high cognitive function throughout the lifespan [47].

It was found that middle-aged and older adults with sarcopenia were associated with low cognitive trajectory, consistent with the results of an analysis by Xue et al. [46]. Cross-lagged analyses showed a bidirectional negative association between sarcopenia and cognitive function. Sensitivity analyses also suggested that there are long-term effects of sarcopenia, showing a significant effect under the interaction of time. Some scholars have suggested reasons from the perspective of mechanisms that sarcopenia can reduce muscle mass and strength, causing an imbalance in myokine secretion, including inflammatory cytokines, apelin, brain-derived neurotrophic factor, and chemokines. This imbalance in myokine secretion further promotes an upregulation of proinflammatory cytokine production through blood–brain barrier transmigration, ultimately resulting in memory impairment [48]. Other research suggests that muscle proteins may reach the central nervous system (CNS) through the systemic circulation, thereby affecting cognitive performance [49]. Evidence further indicates that the effects of neuropathology on motor decline are multifaceted, with some neuropathologies associated with both motor decline and cognitive variability [50]. Conversely, cognitive impairment may reduce physical activity and dietary intake, contributing to accelerated muscle loss and sarcopenia progression in older adults [51]. Therefore, early detection and intervention targeting muscle health may offer dual benefits, preserving both physical function and cognitive abilities in older adults. In addition, adopting a multidisciplinary strategy that combines resistance training, anti-inflammatory dietary patterns, and mental health support may help break the pathological cycle between muscle decline and cognitive deterioration. From a policy perspective, integrating sarcopenia assessments into community-based elderly care programs may serve as an effective means of reducing the long-term burden of cognitive impairment and dementia on healthcare systems.

Depression was associated with low cognitive trajectories, with cross-lagged analyses revealing bidirectional effects between depression and cognitive function. Sensitivity analyses also suggested a significant interaction between depression and time. This is inconsistent with previous research on the relationship between depression and cognitive decline [52,53]. Notably, while there is substantial research on depression trajectories [54,55], the impact of depression on cognitive trajectories in older adults has been reported in only one study among 394 individuals [56]. Our study, therefore, provides support for the evidence through national data exploration.

Mediation analyses indicated a significant mediating effect of depression symptoms between sarcopenia and cognitive function, consistent with the findings of a cross-sectional study by Chen [26]. The findings were validated by two pieces of evidence derived from the longitudinal data analysis. First, baseline results indicated that depressive symptoms mediated the effect of sarcopenia on cognitive trajectories, with the mediating effect accounting for 11.78% of the total effect. This suggested that sarcopenia negatively impacts cognitive performance, partly through its influence on depressive symptoms. Individuals with sarcopenia may experience low mood or depression due to declining physical function, which, in turn, can accelerate cognitive decline. It has been previously highlighted that sarcopenia reduces mobility and self-care, which may increase feelings of loneliness and depression [57]. From a biological perspective, sarcopenia-related increases in inflammatory cytokines may adversely affect the central nervous system, leading to depressive symptoms and further reduced mobility [58]. These depressive symptoms can subsequently impair cognitive function, as evidenced by the increased risk of progression from mild cognitive impairment to Alzheimer’s disease and dementia among individuals with depression [25,59]. Therefore, we recommend mental health support services for middle-aged and older adults with sarcopenia, including management of anxiety, depression, and other mood disorders. Particularly in the early stages of sarcopenia, psychological support may adjust their perceptions of physical changes and reduce the onset of depression.

Secondly, cross-lagged mediation analyses further demonstrated a significant mediating effect in the pathway “T1 cognitive function → T2 depression → T3 sarcopenia”. This suggests that early cognitive decline may lead to increased depression symptoms, which subsequently raise the risk of sarcopenia. Cognitive decline is thought to reduce engagement in physical and social activities [51], contributing to both muscle loss and worsening mood, ultimately elevating sarcopenia risk. Additionally, psychological stress-associated inflammatory responses triggered by cognitive impairment may further exacerbate sarcopenia risk [48]. It is, therefore, recommended that assessments of cognitive function and depression be incorporated into sarcopenia evaluations to provide a more comprehensive view of physical and mental health in middle-aged and older adults, enabling early detection and timely intervention.

In contrast, no significant mediating effect was observed in the pathway “T1 sarcopenia → T2 depression → T3 cognitive function”. This phenomenon may be attributed to the complex interaction between sarcopenia and depression. Firstly, the direct effect of sarcopenia on depression appears to be limited. While sarcopenia leads to muscle loss and physical weakness, primary risk factors for depression in older adults include chronic illness, stressful life events, and poor social support [60]. Sarcopenia, therefore, may not be the determining factor that contributes to depression. Additionally, the indirect effects of sarcopenia on cognitive function likely operate through alternative mechanisms, such as inflammatory markers, metabolic syndrome, and reduced physical activity, rather than solely through depressive symptoms. Future research should, therefore, consider these alternative pathways to better understand the interactions between sarcopenia and cognitive function.

The strengths of this study include the use of a large, nationally representative cohort of middle-aged and older Chinese adults, providing robust insights into the interactions between sarcopenia, depression, and cognitive function. With three waves of follow-up data, we were able to assess changes in cognitive trajectories over time. Advanced statistical methods, including mediation analysis and cross-lagged panel modeling (CLPM), were employed to examine the bidirectional and mediating effects between variables. This approach enabled the identification of distinct cognitive trajectories and provided empirical evidence for the indirect effects of depressive symptoms on sarcopenia risk, adding depth to current knowledge on the complex interplay between cognitive and physical health in aging populations.

This study has several limitations. First, some disease-related data and depressive symptoms were primarily self-reported, which may introduce recall bias and affect the accuracy of these measures. Second, since sarcopenia variables were not measured in 2018 and 2020, this study utilized only three waves of data from 2011 to 2015, which poses certain limitations in terms of data timeliness. Future studies with longer follow-up periods are needed to determine whether the observed relationships between these diseases persist over time. Third, variable selection was limited by the constraints of the available databases. Although multiple covariates were controlled for based on prior knowledge, certain confounders may not have been accounted for. Finally, the potential biological mechanisms underlying these associations could not be explored, underscoring the need for further experimental studies to validate these findings.

## 5. Conclusions

This study highlights the complex interactions between sarcopenia, depression symptoms, and cognitive function in middle-aged and older adults. Our findings identify four cognitive trajectories, with sarcopenia and depression symptoms associated with worse cognitive trajectories over time. Cross-lagged mediation analyses revealed a significant pathway in which early cognitive impairment leads to depressive symptoms, subsequently increasing the risk of sarcopenia. These results suggest that cognitive decline can exacerbate sarcopenia indirectly through its impact on mental health, likely due to reduced physical and social activities and the associated inflammatory responses. However, the pathway from depression-mediated sarcopenia to cognitive function was not significant, indicating that sarcopenia’s impact on cognitive function may involve other physiological mechanisms beyond depressive symptoms. These findings underscore the importance of early screening for both cognitive impairment and depression symptoms in sarcopenia assessments, facilitating timely intervention and a more comprehensive approach to managing physical and mental health in aging populations. Future research should continue to explore the multifaceted mechanisms linking sarcopenia and cognitive function, particularly examining other biological and behavioral pathways.

## Figures and Tables

**Table 1:** Fitting statistics for cognitive function score trajectories by GBTM.

Variable	Polynomial Order by Group					
	1	2	3	4	5	6
	−1	(1, 2)	(1, 1, 2)	(1, 1, 1, 2)	(1, 1, 1, 1, 2)	(1, 2, 2, 2, 1, 2)
BIC (*n* = 7091)	−54,983.53	−51,959.34	−51,282.61	−51,161.54	−51,091.78	−51,034.94
AIC	−54,973.23	−51,935.31	−51,248.28	−51,116.91	−51,036.85	−50,959.41
Proportion						
Group 1	1.00	28.39	12.18	5.90	6.33	7.02
Group 2		71.61	37.27	18.62	7.12	7.87
Group 3			50.55	38.76	12.75	5.79
Group 4				36.73	39.11	8.52
Group 5					34.69	39.00
Group 6						31.80
AvePP						
Group 1	—	0.91	0.89	0.85	0.83	0.84
Group 2		0.96	0.84	0.79	0.66	0.64
Group 3			0.90	0.76	0.69	0.65
Group 4				0.85	0.76	0.65
Group 5					0.84	0.76
Group 6						0.84

**Table 2:** Baseline characteristics of participants based on trajectories of cognitive scores.

Baseline Characteristics	Overall	Low and Decline	Middle and Decline	Middle and Stable	High and Stable	*x*^2^/*F*
	(*n* = 7091)	(*n* = 388)	(*n* = 1280)	(*n* = 2860)	(*n* = 2563)	
Age, *n* (%)						
45–59	4156 (58.61)	145 (3.49)	631 (15.18)	1643 (39.53)	1737 (41.79)	266.419 ***
60–74	2717 (38.32)	203 (7.47)	587 (21.60)	1139 (41.92)	788 (29.00)	
≥75	218 (3.07)	40 (18.35)	62 (28.44)	78 (35.78)	38 (17.43)	
Gender, *n* (%)						124.372 ***
Male	3832 (54.04)	145 (3.78)	564 (14.72)	1620 (42.28)	1503 (39.22)	
Female	3259 (45.96)	243 (7.46)	716 (21.97)	1240 (38.05)	1060 (32.53)	
Residence, *n* (%)						585.923 ***
Rural	5354 (75.54)	352 (6.57)	1140 (21.29)	2334 (43.59)	1528 (28.54)	
Urban	1734 (24.46)	36 (2.08)	139 (8.02)	524 (30.22)	1035 (59.69)	
Educational level, *n* (%)						1547.554 ***
Primary school or below	4137 (58.37)	370 (8.94)	1118 (27.02)	1834 (44.33)	815 (19.70)	
Junior high school	1892 (26.69)	13 (0.69)	130 (6.87)	753 (39.80)	996 (52.64)	
Senior high school or above	1059 (14.94)	5 (0.47)	31 (2.93)	272 (25.68)	751 (70.92)	
Marital status, *n* (%)						109.779 ***
Married	6467 (91.20)	315 (4.87)	1107 (17.12)	2642 (40.85)	2403 (37.16)	
Others	624 (8.80)	73 (11.70)	173 (27.72)	218 (34.94)	160 (25.64)	
Medical insurance, *n* (%)						6.612
Yes	6710 (94.84)	358 (5.34)	1200 (17.88)	2713 (40.43)	2439 (36.35)	
No	365 (5.16)	27 (7.40)	76 (20.82)	147 (40.27)	115 (31.51)	
Sleep, mean (SD)	6.4 (1.7)	276 (6.2)	852 (19.1)	1807 (40.6)	1515 (34)	21.093 ***
Smoke, *n* (%)						19.001 ***
Yes	3068 (43.27)	149 (4.86)	500 (16.30)	1303 (42.47)	1116 (36.38)	
No	4022 (56.73)	238 (5.92)	780 (19.39)	1557 (38.71)	1447 (35.98)	
Drink, *n* (%)						34.383 ***
Yes	2641 (37.24)	112 (4.24)	428 (16.21)	1053 (39.87)	1048 (39.68)	
No	4450 (62.76)	276 (6.20)	852 (19.15)	1807 (40.61)	1515 (34.04)	
BMI, *n* (%)						117.134 ***
Normal	3173 (50.95)	214 (6.74)	633 (19.95)	1321 (41.63)	1005 (31.67)	
Underweight	212 (3.40)	26 (12.26)	65 (30.66)	88 (41.51)	33 (15.57)	
Overweight	2017 (32.39)	79 (3.92)	332 (16.46)	819 (40.60)	787 (39.02)	
Obese	826 (13.26)	33 (4.00)	127 (15.38)	332 (40.19)	334 (40.44)	
Number of chronic diseases, *n* (%)						21.282 **
0	2271 (32.03)	105 (4.62)	402 (17.70)	874 (38.49)	890 (39.19)	
1	2057 (29.01)	105 (5.10)	376 (18.28)	839 (40.79)	737 (35.83)	
≥2	2763 (38.96)	178 (6.44)	502 (18.17)	1147 (41.51)	936 (33.88)	
ADL, *n* (%)						171.642 ***
Yes	899 (12.79)	100 (11.12)	238 (26.47)	378 (42.05)	183 (20.36)	
No	6132 (87.21)	287 (4.68)	1028 (16.76)	2462 (40.15)	2355 (38.41)	
IADL, *n* (%)						232.434 ***
Yes	1042 (14.69)	122 (11.71)	276 (26.49)	443 (42.51)	201 (19.29)	
No	6049 (85.31)	266 (4.40)	1004 (16.60)	2417 (39.96)	2362 (39.05)	
Pain, *n* (%)						172.275 ***
Yes	2139 (30.19)	174 (8.13)	487 (22.77)	924 (43.20)	554 (25.90)	
No	4946 (69.81)	213 (4.31)	793 (16.03)	1935 (39.12)	2005 (40.54)	
SRH, *n* (%)						237.228 ***
Very good	500 (7.06)	14 (2.80)	69 (13.80)	194 (38.80)	223 (44.60)	
Good	1311 (18.50)	48 (3.66)	172 (13.12)	485 (36.99)	606 (46.22)	
Normal	3650 (51.50)	172 (4.71)	660 (18.08)	1479 (40.52)	1339 (36.68)	
Poor	1390 (19.61)	126 (9.06)	314 (22.59)	604 (43.45)	346 (24.89)	
Very poor	236 (3.33)	28 (11.86)	64 (27.12)	96 (40.68)	48 (20.34)	

Note: ** indicates *p* value < 0.01; *** indicates *p* value < 0.001.

**Table 3:** Description of sarcopenia, depression, and cognitive function scores.

Baseline Characteristics	Sarcopenia, *n* (%)		CED-10, Mean ± SD	Depression Symptom, *n* (%)		Cognitive Function Scores (MMSE), Mean ± SD
	Yes	No		Yes	No	
Wave						
2011	373 (5.26)	6718 (94.74)	7.60 ± 5.979	2240 (31.59)	4851 (68.41)	12.615 ± 3.101
2013	351 (4.95)	6740 (95.05)	7.31 ± 5.455	1983 (27.97)	5108 (72.03)	12.530 ± 3.230
2015	461 (6.50)	6630 (93.50)	7.44 ± 6.076	2158 (30.43)	4933 (69.57)	12.229 ± 3.284
*x*^2^/*F*	18.166		4.252	23.147		28.314
*p*	<0.001		0.014	<0.001		<0.001
Cognitive trajectory						
Low and decline	63 (16.89)	325 (4.84)	9.61 ± 6.535	216 (9.64)	172 (3.55)	6.914 ± 2.234
Middle and decline	113 (30.29)	1167 (17.37)	7.85 ± 5.891	562 (25.09)	718 (14.80)	9.633 ± 2.27
Middle and stable	153 (41.02)	2707 (40.29)	5.76 ± 4.994	958 (42.77)	1902 (39.21)	12.422 ± 2.008
High and stable	44 (11.80)	2519 (37.50)	7.60 ± 5.979	504 (22.50)	2059 (42.44)	15.182 ± 1.67
*x*^2^/*F*	191.131		192.512	367.436		3560.884
*p*	<0.001		<0.001	<0.001		<0.001

## Data Availability

The data supporting the findings of this study are available from the corresponding author upon reasonable request. The data are not publicly available due to privacy considerations.

## Supplementary Materials

The following supporting information can be downloaded at: https://www.mdpi.com/article/10.3390/brainsci15040408/s1, Figure S1: Cross-lagged relationship between sarcopenia and cognitive impairment; Figure S2: Trajectories of episodic memory and mental intactness scores; Table S1: Mediation effect of depression on the relationship between sarcopenia and cognitive trajectories/ Cognitive function; Table S2: Correlation analysis of sarcopenia, depressive score and cognitive function score in middle-aged and elderly people; Table S3: The model fit information of different models; Table S4: Fitting statistics for episodic memory and mental intactness scores trajectories.

## List of Abbreviations

MCI	Mild cognitive impairment
AD	Alzheimer’s disease
AWGS	Asian Working Group for Sarcopenia
ASM	Appendicular skeletal muscle mass
BMI	Body mass index
ADL	Activities of Daily Living
IADL	Instrumental Activities of Daily Living
SRH	Self-rated health
GBTM	Group-based trajectory modeling
BIC	Bayesian Information Criterion
AIC	Akaike information criterion
AvePP	Average posterior probability
CLPM	Cross-lagged panel modeling
