# Supplementary material for: Longitudinal Trajectories of Cognitive Function Among Chinese Middle-Aged and Older Adults: The Role of Sarcopenia and Depressive Symptoms
Source: Brain Sci. 2025 Apr 17;15(4):408. doi: 10.3390/brainsci15040408 (PMC12025789; doi:10.3390/brainsci15040408)
Supplement: Supplementary file 1 [file brainsci-15-00408-s001.zip › brainsci-3575456-supplementary.pdf]

Longitudinal trajectories of cognitive function among Chinese middle-aged and older adults: the role of sarcopenia and depressive symptoms

Supplementary Data List:

Figure S1 Cross-lagged relationship between sarcopenia and cognitive impairment
Figure S2 Trajectories of episodic memory and mental intactness scores

Table S1 Mediation effect of depression on the relationship between sarcopenia and cognitive trajectories/ Cognitive function.
Table S2: Correlation analysis of sarcopenia, depressive score and cognitive function score in middle-aged and elderly people
Table S3: The model fit information of different models
Table S4: Fitting statistics for episodic memory and mental intactness scores trajectories

Figure

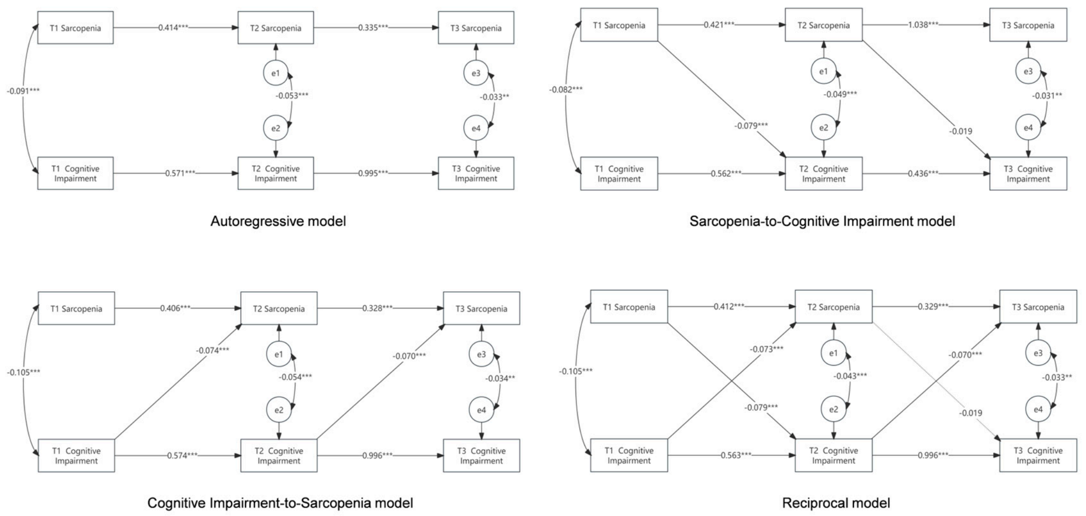

Supplement Figure S1 Cross-lagged relationship between sarcopenia and cognitive impairment

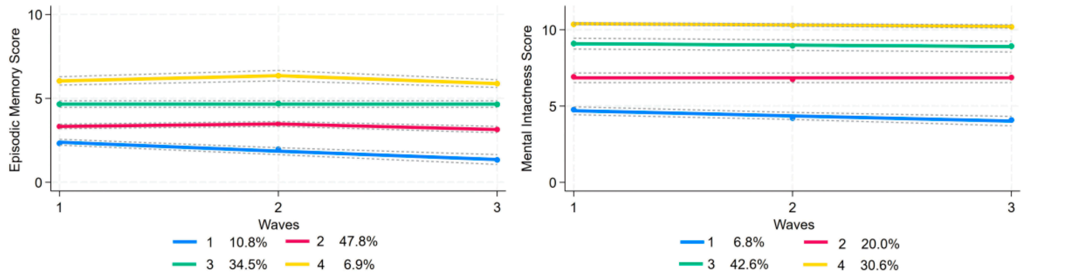

Supplement Figure S2 Trajectories of episodic memory and mental intactness scores by GBTM

Table

Supplement Table S1 Mediation effect of depression on the relationship between sarcopenia and cognitive trajectories/ Cognitive function

Effection	Path	Effect value/ β	*P*-Value	95% CI
Cognitive trajectories				
Direct effect	Sarcopenia → Cognitive trajectories	-0.5534	<0.001	(-0.6441, -0.4600)
Indirect effect	Sarcopenia →Depressive symptoms → Cognitive trajectories	-0.0739	<0.001	(-0.0961, -0.0500)
Total effect	Sarcopenia → Cognitive trajectories	-0.6273	<0.001	(-0.7220, -0.5300)
Cognitive function				
Direct effect	T1 Cognitive function → T3 Sarcopenia	-0.0622	0.001	(-0.0973, -0.0271)
Indirect effect	T1 Cognitive function → T2 Depression → T3 Sarcopenia	-0.0035	0.001	(-0.0056, -0.0014)
Total effect	T1 Cognitive function → T3 Sarcopenia	-0.0657	<0.001	(-0.1026,- 0.0288)
Path	T1 Cognitive function → T2 Depression	-0.2815	<0.001	(-0.3274, -0.2356)

coefficients				
	T2 Depression → T3 Sarcopenia	0.0561	<0.001	(0.0388, 0.0734)

Supplement Table S2: Correlation analysis of sarcopenia, depressive score and cognitive function score in middle-aged and elderly people

Variables	1	2	3	4	5	6	7	8	9
1 T1 Sarcopenia	1.000								
2 T1 Depressive score	-0.119***	1.000							
3 T1 Cognitive function score	0.102***	-0.279***	1.000						
4 T2 Sarcopenia	0.421***	-0.117***	0.084***	1.000					
5 T2 Depression score	-0.122***	0.557***	-0.234***	-0.122***	1.000				
6 T2 Cognitive function score	0.078***	-0.197***	0.466***	0.057***	-0.230***	1.000			
7 T3 Sarcopenia	0.440***	-0.119***	0.105***	0.457***	-0.127***	0.081***	1.000		
8 T3 Depression score	-0.152***	0.557***	-0.224***	-0.138***	0.598***	-0.189***	-0.152***	1.000	
9 T3 Cognitive function score	0.073***	-0.222***	0.451***	0.074***	-0.211***	0.511***	0.092***	-0.248***	1.000

***P < 0.001

Supplement Table S3: The model fit information of different models by CLPA

Model	CFI	TLI	RMSEA	SRMR	x^2	df
Model 1	0.963	0.913	0.066	0.055	193.987	6
Model 2	0.974	0.909	0.068	0.038	135.409	4
Model 3	0.978	0.924	0.062	0.032	113.965	4
Model 4	0.995	0.962	0.044	0.012	29.230	2
XY-Z	0.997	0.950	0.045	0.010	30.276	2
XMY	0.983	0.908	0.080	0.030	281.074	6
XMY-Z	0.993	0.924	0.059	0.016	151.786	6

Supplement Table S4: Fitting statistics for episodic memory and mental intactness scores trajectories by GBTM

Variable	Episodic memory scores					Mental intactness scores				
	1	2	3	4	5	1	2	3	4	5
	1	(2 1)	(1 1 1)	(1 0 1 1)	(2 1 1 1 1)	1	(2 2)	(1 2 2)	(1 2 0 2)	(1 2 1 2 2)
BIC	-44596.47	-42368.87	-41940.41	**-41886.64**	-41874.91	-40928.90	-39561.12	-39247.56	**-39200.48**	-39194.22
AIC	-44586.17	-42344.84	-41909.51	**-41848.88**	-41819.98	-40918.60	-39533.65	-39209.79	**-39155.84**	-39132.42
Proportion										
Group 1	1.000	28.067	11.756	**6.776**	6.757	1.000	53.542	18.720	**10.791**	10.560
Group 2		71.933	39.575	**19.979**	18.393		46.458	59.76177	**47.793**	45.657
Group 3			48.669	**42.624**	1.843			21.518	**34.534**	1.374
Group 4				**30.622**	42.481				**6.882**	35.347
Group 5					30.527					7.062
Group 6										
AvePP										
Group 1	—	0.888	0.858	**0.835**	0.836		0.875	0.802	**0.786**	0.784
Group 2		0.953	0.825	**0.753**	0.723		0.868	0.829	**0.770**	0.761
Group 3			0.865	**0.738**	0.651			0.820	**0.729**	0.639
Group 4				**0.763**	0.743				**0.751**	0.742
Group 5					0.774					0.761
